# Gate-Controlled Supercurrent in Epitaxial Al/InAs Nanowires

**DOI:** 10.1021/acs.nanolett.1c03493

**Published:** 2021-11-02

**Authors:** Tosson Elalaily, Olivér Kürtössy, Zoltán Scherübl, Martin Berke, Gergö Fülöp, István Endre Lukács, Thomas Kanne, Jesper Nygård, Kenji Watanabe, Takashi Taniguchi, Péter Makk, Szabolcs Csonka

**Affiliations:** † Department of Physics and Nanoelectronics “Momentum” Research Group of the Hungarian Academy of Sciences, Budapest University of Technology and Economics, Budafoki ut 8, 1111 Budapest, Hungary; ‡ Department of Physics, Faculty of Science, Tanta University, Al-Geish Street, 31527 Tanta, Gharbia, Egypt; ¶ Université Grenoble Alpes, CEA, Grenoble INP, IRIG, PHELIQS, 38000 Grenoble, France; ⊥ Center for Energy Research, Institute of Technical Physics and Material Science, Konkoly-Thege Miklós út 29-33., H-1121 Budapest, Hungary; # Center for Quantum Devices and Nano-Science Center, Niels Bohr Institute, University of Copenhagen, Universitetsparken 5, DK-2100 Copenhagen, Denmark; ∇ Research Center for Functional Materials, National Institute for Material Science, 1-1 Namiki, Tsukuba 305-0044, Japan; ○ International Center for Materials Nanoarchitectonics, National Institute for Materials Science, 1-1 Namiki, Tsukuba 305-0044, Japan

**Keywords:** field effect, epitaxial superconductors, nanowire, gate-controlled supercurrent, hot electron injection, phonons

## Abstract

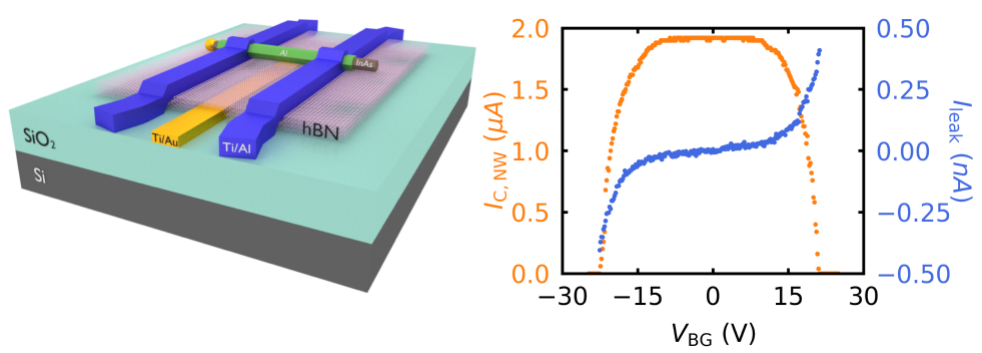

Gate-controlled supercurrent (GCS) in superconducting nanobridges has recently attracted attention as a means to create superconducting switches. Despite the clear advantages for applications, the microscopic mechanism of this effect is still under debate. In this work, we realize GCS for the first time in a highly crystalline superconductor epitaxially grown on an InAs nanowire. We show that the supercurrent in the epitaxial Al layer can be switched to the normal state by applying ≃±23 V on a bottom gate insulated from the nanowire by a crystalline hBN layer. Our extensive study of the temperature and magnetic field dependencies suggests that the electric field is unlikely to be the origin of GCS in our device. Though hot electron injection alone cannot explain our experimental findings, a very recent non-equilibrium phonons based picture is compatible with most of our results.

## Introduction

Superconducting circuits have become promising building blocks in various architectures for quantum computing devices,^1,2^ single photon detectors,^3,4^ quantum-limited amplifiers,^5^ phase-coherent caloritronics,^6,7^ ultrasensitive magnetometers^8,9^ and fast classical supercomputers.^10,11^ In the latter, the superconducting electronics are integrated with semiconductor technology. In particular, rapid single flux quantum (RSFQ) devices become more desirable than semiconducting switches due to their fast switching speed and low power consumption.^10^ Since RSFQ consists of a flux-controlled superconducting loop with Josephson junctions along with on-chip coils, its upscaling remains a challenge. In order to realize scalable superconducting switches, electrical control via gate electrodes would be desirable.

Very recently a striking new effect was observed in metallic nanostructures. In superconducting nanobridges the supercurrent can be controlled by applying a voltage on a closely spaced gate electrode.^12−24^ By increasing the gate voltage beyond a certain threshold, the supercurrent in the nanobridge can be quenched. Previous works show GCS in thin metallic nanowires,^12,18,22,23^ in proximitized normal metal in a superconductor–normal–superconductor (SNS) junction^13^ and in Dayem nanobridges.^15,17,20^ In addition to high-speed superconducting switches,^23^ observation of GCS in superconducting nanostructures led to the realization of new nanodevices such as gate-controlled superconducting phase shifter^16^ and half-wave nanorectifiers.^20^ Despite the clear advantages of GCS for applications, the origin of the effect is still under debate. Some works explained the suppression of the supercurrent by an electric field induced perturbation of the superconducting state,^25−27^ such as, e.g., by Swinger effect,^28^ others attributed the effect to injection of high-energy quasiparticles tunneling from the gate electrode.^22−24^ Even though the microscopic origin of the effect is unclear, gate-controlled nanobridges can work at ultralow power and high switching speed. In addition, their configuration can be easily scaled up, which provides a promising building block for superconducting switches in modern architectures of both classical and quantum computers.

Recently InAs semiconductor nanowires with epitaxial Al superconducting shells^29^ have become the primary platform for research on various potential quantum devices. Concepts have been developed for topologically protected qubits^30^ and surface code for majorana states^31^ based on hybrid nanowires, and they are also promising for realization of gatemon or Andreev qubits.^32,33^ In all of these examples of quantum hardware, the gate tunable superconductivity would be highly desirable to provide an additional experimental control knob of the system. However, up to now, all gate tuning experiments were performed on polycrystalline materials and therefore it has not been clear if GCS exists for highly crystalline materials. In this work, we studied for the first time the superconducting gating effect in highly crystalline Al shells epitaxially grown on InAs nanowires.^29^ We will show in the following that the superconducting state can be switched off by applying voltage on a nearby metallic electrode and provide detailed characteristics of the gating behavior.

## Results and Discussion

We have used InAs nanowires grown by molecular beam epitaxy (MBE) using gold nanoparticles as catalysts. After InAs nanowires growth, an Al shell layer of thickness 20 nm was epitaxially grown by deposition within the MBE chamber at low temperature. By rotating the substrate during Al growth, the Al shell layer is grown in all InAs nanowire facets, resulting in fully covered nanowires.^29^

**Figure 1:**
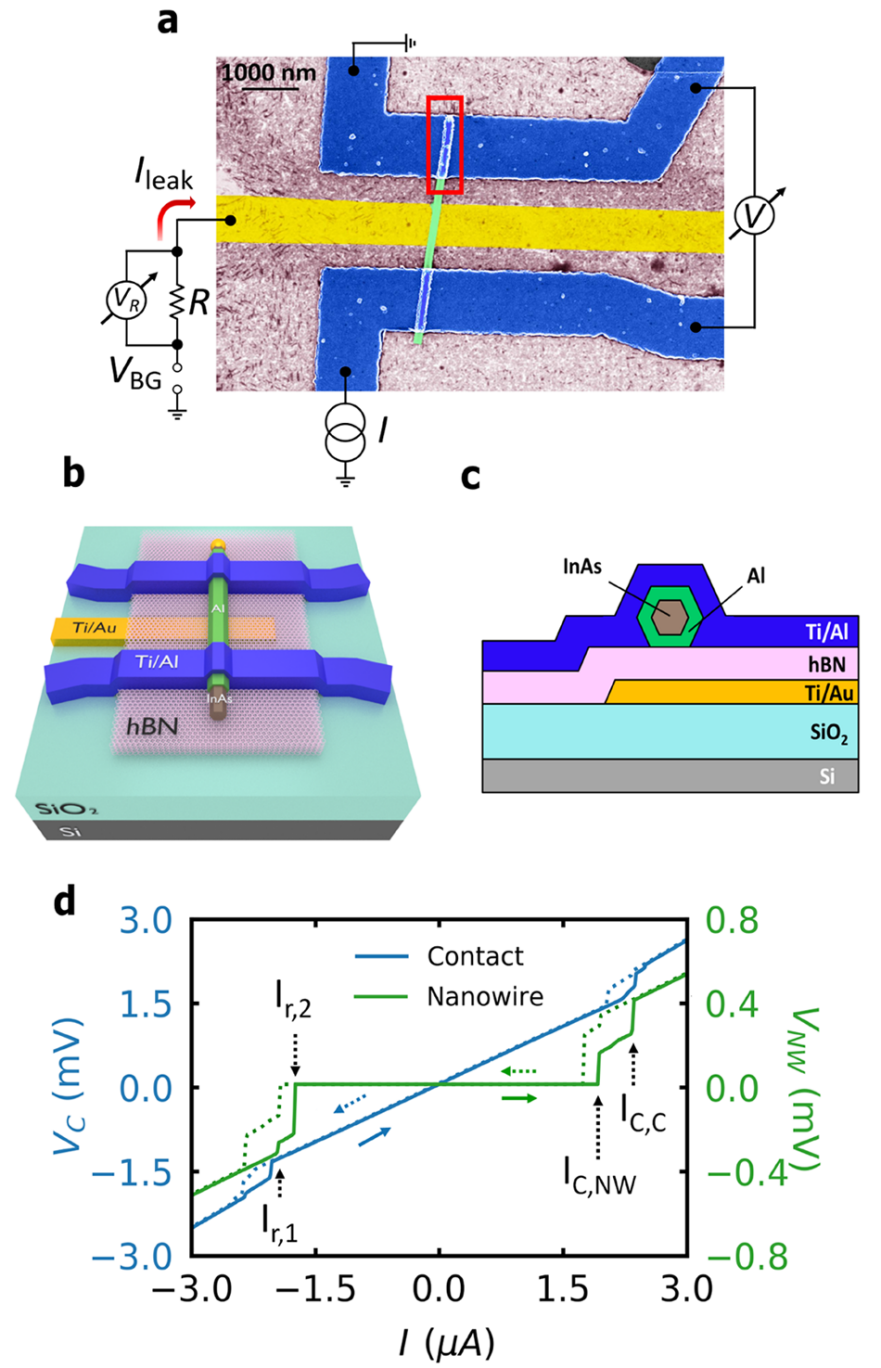
Device configuration. (a) False colored SEM image of the fabricated device with schematics of the device circuit. Schematic of the device with (b) 45° angle view and (c) side view. (d) *I*–*V* characteristics of the nanowire device (green curve) at 40 mK with two different switchings at *I*_C,NW_ and *I*_C,C_ in the case of bias current is ramped from negative to positive values (solid green arrow). In the opposite ramping direction (dotted green arrow), it switches back at two successive retrapping current values, *I*_r,1_ and *I*_r,2_. Measurement of the top pair of Al contacts using the two-probe method (blue curve), showing the switching of the contacts at the same value of *I*_C,C_.

To investigate the gate-controlled supercurrent in InAs nanowires with epitaxial superconducting layer, first, we fabricated devices with the standard geometry using side gates^12−23^ or a backagte,^12^ where the gating effect was observed in eight devices. In order to improve the geometry, i.e., minimize the separation between the gate and the nanowire and keep the leakage current small, we developed the geometry presented in Figure 1a–c. A metallic gate from Ti/Au (yellow) with thicknesses of 7/33 nm was fabricated on an intrinsic Si wafer with a 290 nm thick oxide layer. To insulate the gate from the wire, 20–30 nm thick hBN (pink) was stacked on the gate electrode with PDMS-based dry transfer technique. hBN is an excellent single crystal insulator between the gate and the wire, serving as a tunnel barrier.^34−38^ The nanowire (gray) with Al shell (green) was deposited by a micromanipulator on top of the hBN layer. Two pairs of Al contacts (blue) have been fabricated on the top of the nanowire with a distance of 1.5 μm to allow quasi-four-probe measurements. More details about fabrication are given in Methods (see the Supporting Information).

The current–voltage (*I*–*V*) characteristics of the nanowire device measured at 40 mK clearly show a well developed zero resistance state (see green curve in Figure 1d) corresponding to a supercurrent flowing through the Al shell of the wire. Two clear switches to a finite resistance state are observed at 1.94 and 2.34 μA. Similar multiple transitions were observed in a suspended Ti nanowire.^18^ The nanowire device shows a hysteretic behavior and switches back at two successive retrapping current values at *I*_r,1_ = 1.94 μA and *I*_r,2_ = 1.74 μA when the measurements were carried out in the opposite ramping direction. To identify the origin of the two switching steps, we have separately measured the *I*–*V* curves for each horizontal pair of contacts (blue electrodes in Figure 1a) using a two-probe method. The measurements for the top pair with blue curve switch at 2.34 μA, while the bottom electrodes switch at 7.5 μA (see the Supporting Information). From this, we could attribute the switching at the lower current to the SC-normal transition of the epitaxial Al shell, *I*_C,NW_, while the switching at the higher current is the same as that of the Al contact segment above the nanowire, *I*_C,C_, which is marked by the red rectangle in Figure 1a.

**Figure 2:**
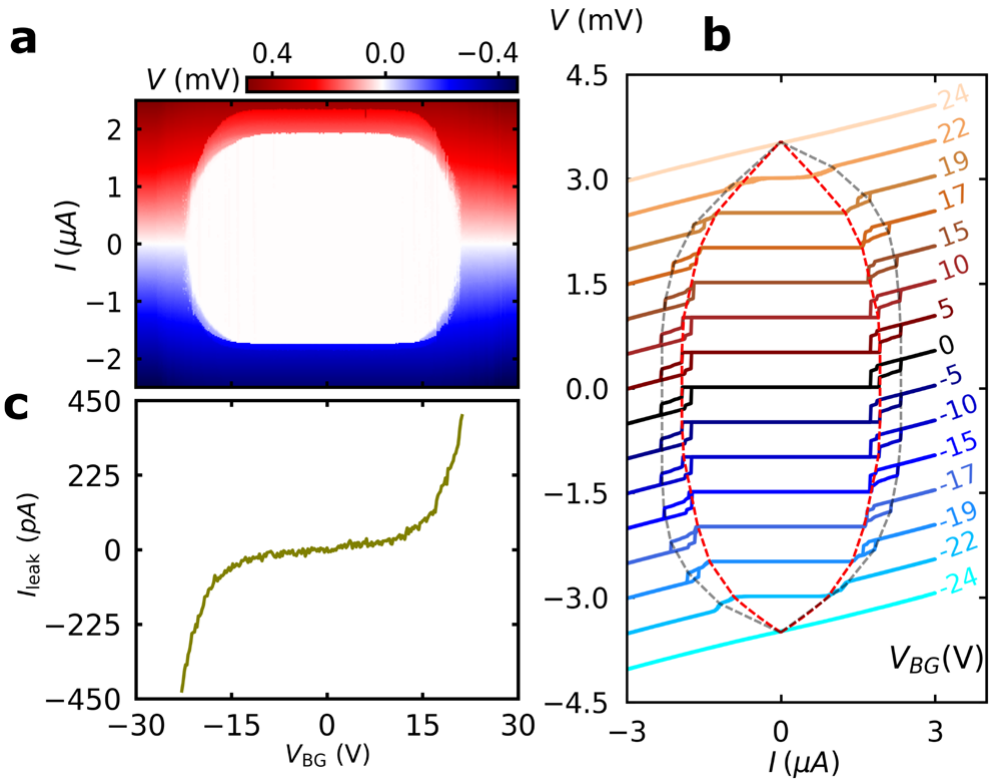
Gating of supercurrent. (a) *I*–*V* characteristics of the nanowire device as a function of bipolar voltage applied to the bottom gate, *V*_BG_, as the current was sweeping from negative to positive values. (b) High-resolution *I*–*V* curves measured at selected gate voltages and equally separated on *y*-axis for better visibility. The red and gray dashed lines trace the suppression of *I*_C,NW_ and *I*_C,C_ with increasing *V*_BG_, respectively. (c) Measured and corrected leakage current from bottom gate to nanowire device as a function of *V*_BG_.

The dependence of the supercurrent on the gate voltage was investigated by measuring the *I*–*V* curve of the nanowire device as a function of the bottom gate voltage, *V*_BG_ (see Figure 2a). The white regions represent the zero resistance state. With increasing *V*_BG_ with either negative or positive polarity, both *I*_C,NW_ and *I*_C,C_ remain constant. Beyond the threshold at *V*_BG_ ≃ ±12 V, both critical currents are suppressed together up to full suppression at the critical gate voltage, *V*_BGC_ ≃ ±23 V, at which the device is switched to the normal state. The maximum electric field estimated at the critical gate voltage is *E*_max_ ≈ 200 MVm^–1^, which is in the same order of magnitude as that reported in refs^18 and 39^. The fine characteristics of the *I*–*V* curves are better visible in Figure 2b, where the red and gray dashed lines trace the suppression of *I*_C,NW_ and *I*_C,C_ with increasing *V*_BG_, respectively. We note that with increasing *V*_BG_, the difference between the retrapping current *I*_r,2_ and *I*_C,C_ is decreased and fully vanished at *V*_BG_ ≈ ±22 V.

The leakage current, *I*_leak_, from the gate to the nanowire device was also measured within a ±*V*_BGC_ window by recording the voltage across the pre-resistor on the gate (see Figure 1a). The leakage current was then corrected by subtracting the leakage between lines of the cryostat (see Supporting Information).^23^ The measured *I*_leak_ shows an exponential increase with *V*_BG_ for both polarities (see Figure 2c), noting that, a strong increase is seen in the leakage current around the gate voltages, where the supercurrents get suppressed.

**Figure 3:**
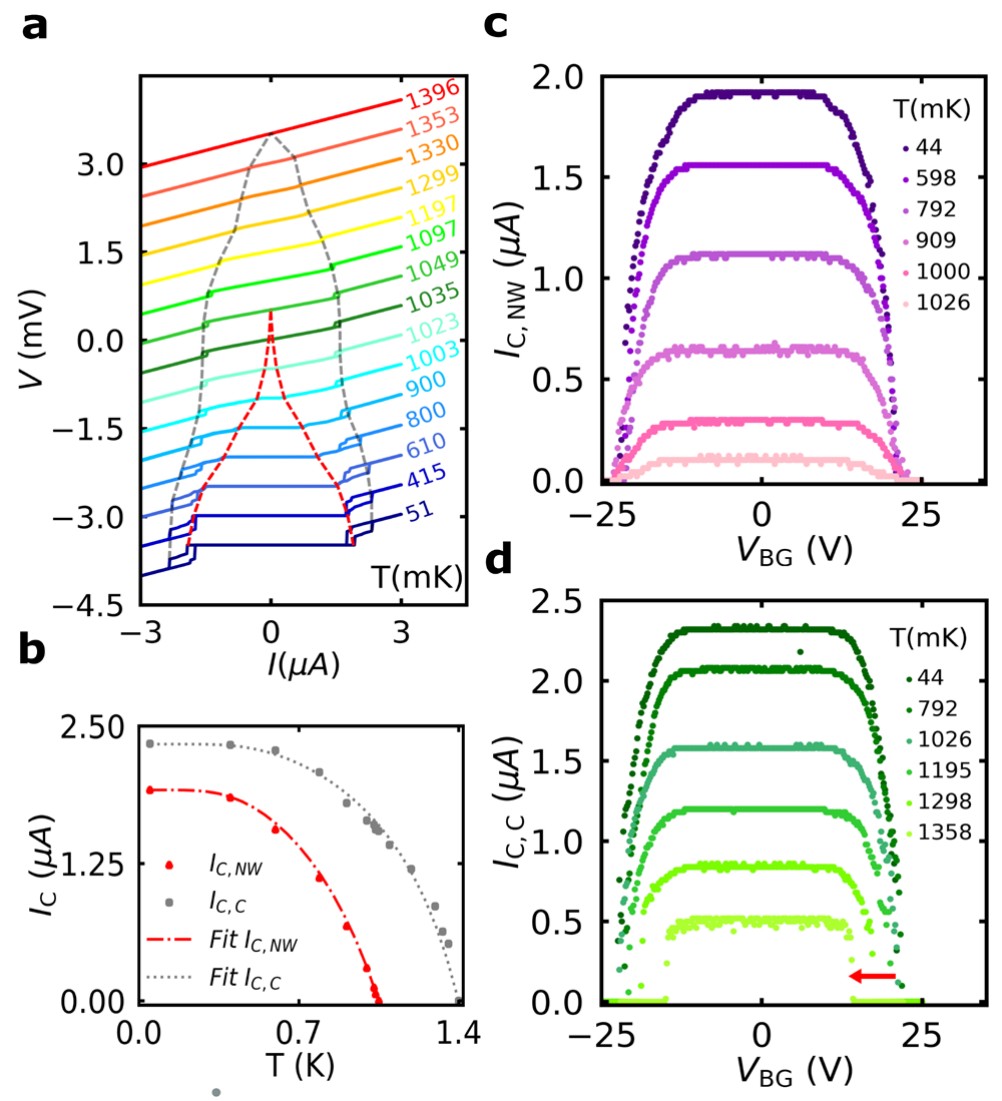
Temperature dependence. (a) *I*–*V* characteristics of the nanowire device at elevated temperatures up to 1400 mK and equally separated on *y*-axis for better visibility. Red and gray dashed lines trace the suppression of both *I*_C,NW_ and *I*_C,C_ with increasing temperature, respectively. *I*_C,NW_ is fully quenched at critical temperature *T*_C,NW_ ≃ 1050 mK, while *I*_C,C_ at *T*_C,C_ ≃ 1400 mK. (b) Temperature dependence of *I*_C,NW_ and *I*_C,C_ extracted from panel a. Temperature dependence of the critical currents is fitted by using Ambegaokar–Baratoff relation illustrated by the red dashed–dotted and the gray dotted lines for *I*_C,NW_ and *I*_C,C_, respectively. (c, d) Critical current as a function of bipolar gate voltage for both *I*_C,NW_ and *I*_C,C_ at elevated temperatures up to 98% of their critical temperatures, respectively.

The critical temperature was determined by measuring the *I*–*V* characteristics of the device at different elevated temperatures at *V*_BG_ = 0 (see Figure 3a). In the case of *I*_C,NW_, it is fully quenched at *T*_C,NW_ ≃ 1050 mK, while for *I*_C,C_ at *T*_C,C_ ≃ 1400 mK with corresponding normal state resistances *R*_n,NW_ = 135 Ω and *R*_n,C_ = 191 Ω, respectively. By extracting the values of *I*_C,NW_ and *I*_C,C_, the dependence of the critical currents on temperature is plotted in Figure 3b. The red dashed–dotted and gray dotted curves are fits of the temperature dependences of *I*_C,NW_ and *I*_C,C_, respectively, by using the Ambegaokar–Baratoff relation:^40−42^
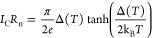
1where
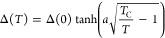
2is the superconducting gap at temperature *T*,^43−45^*R*_n_ is the normal-state resistance, and *k*_B_ is Boltzmann constant. The temperature dependences of both *I*_C,NW_ and *I*_C,C_ are fitted using the coefficient *a* = 2 and 2.4 and *R*_n_ = 130 and 143 Ω, respectively. The latter values of the normal-state resistances are in good agreement with our experimental findings.

The temperature dependence of GCS has been investigated by measuring the critical currents, *I*_C,NW_ and *I*_C,C_ as a function of bipolar gate voltage at elevated temperatures (see Figure 3c,d, respectively). With increasing of the bath temperature, both *I*_C,NW_ and *I*_C,C_ at zero gate voltage respect the temperature dependence of critical currents shown in Figure 3b. However, the gating characteristics look quite similar at all temperatures. The critical gate voltage, *V*_BGC_, in the case of *I*_C,NW_ did not change with increasing *T* up to close to its critical temperature at *T*_C,NW_, while in the case of *I*_C,C_, it only shifts to lower values (indicated by red arrow) for measurements at temperatures higher than *T*_C,NW_. A similar shift of *V*_BGC_ with increasing temperature was observed in ref (18). We have also plotted the critical currents as a function of leakage current for different temperatures (see the Supporting Information). A small change in the critical leakage current is visible due to fluctuations in the leakage current.

**Figure 4:**
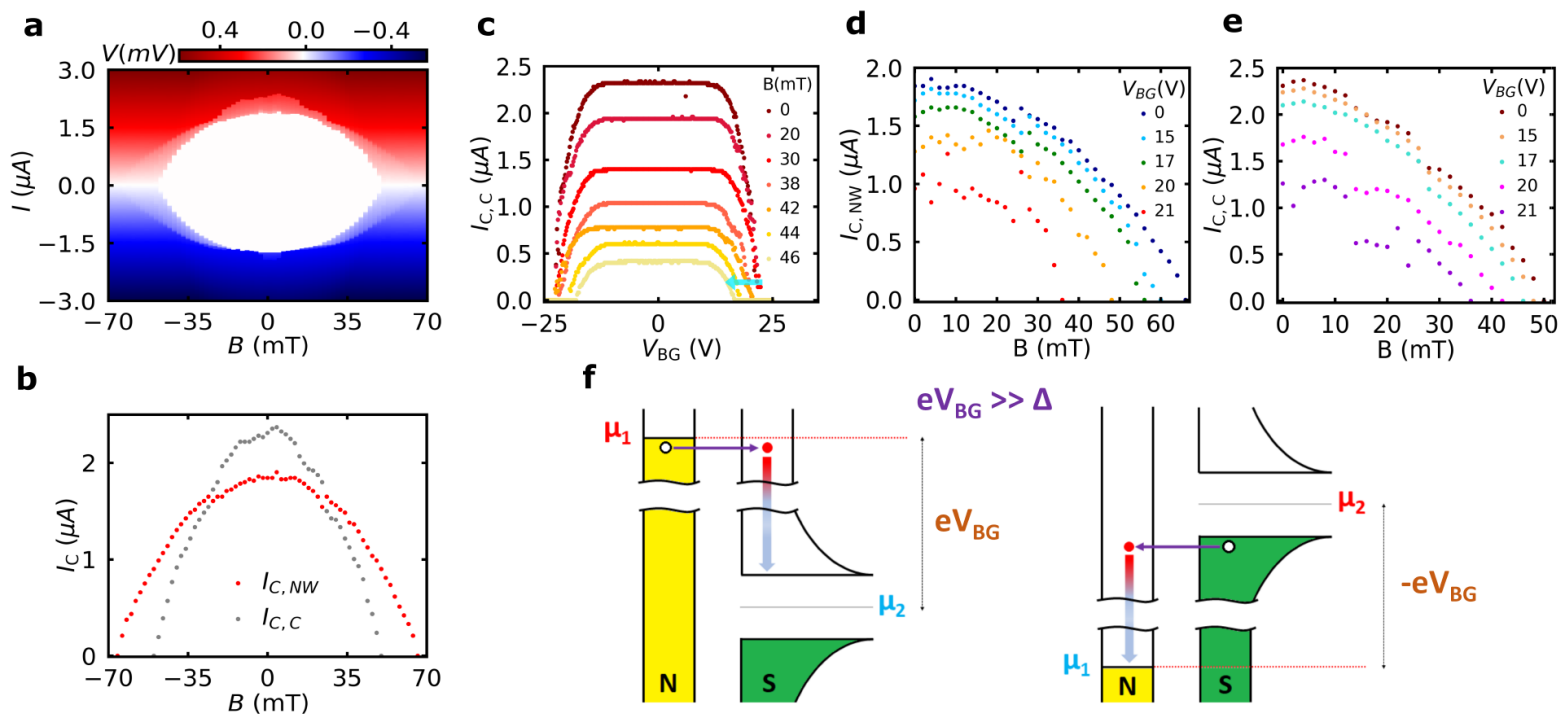
Magnetic field dependence and schematic of ballistic hot electron injection. (a) *I*–*V* characteristics of the nanowire device as a function of out of plane magnetic field. (b) Critical currents of the nanowire device and the contact interface region as a function of the applied magnetic field, extracted from the measurements in panel a. *I*_C,NW_ and *I*_C,C_ cross each other at *B* ≃ ±24 mT, leading to corresponding critical magnetic fields, *B*_C,NW_ = 66 mT and *B*_C,C_ = 50 mT, respectively. (c) *I*_C,C_ as a function of bipolar gate voltage at magnetic fields up to 46 mT. (d, e) *I*_C,NW_ and *I*_C,C_ as a function of magnetic field at gate voltage values close to the critical gate voltage, respectively. (f) Schematic diagrams of ballistic electron injection from/to the metallic gate N to/from the superconducting nanowire device S in the left and right panels, respectively. Colored/uncolored parts represent occupied/unoccupied states. As the hot electron (red circle) tunnels, it will relax to the lowest unoccupied state, releasing heat on either the N or S side, resulting in different heating of the S side as the polarity of *V*_BG_ changes.

The dependence of critical currents with magnetic field was investigated by measuring *I*–*V* characteristics of the nanowire device as a function of out of plane magnetic field, *B*, as shown in Figure 4a. Both *I*_C,NW_ and *I*_C,C_ decrease in the magnetic field, as expected. Moreover, it can be also seen that *I*_C,NW_ and *I*_C,C_ cross each other at *B* ≃ ±24 mT, and their corresponding critical fields are *B*_C,NW_ = 66 mT and *B*_C,C_ = 50 mT, respectively. This is clearly seen in Figure 4b, which shows the magnetic field dependence of critical currents extracted from measurements in Figure 4a.

The dependence of GCS in the nanowire device in finite magnetic field shows a similar dependence as its temperature dependence: that the gate dependence has the same general trend at all magnetic fields up to 46 mT as shown in Figure 4c in the case of *I*_C,C_. On the other hand, *V*_BGC_ decreases when *B* values approach *B*_C,C_. In the same way, *I*_C,NW_ shows a similar magnetic field dependence (see the Supporting Information). We have also measured both critical currents, *I*_C,NW_ and *I*_C,C_, as a function of the magnetic field at different gate voltages up to values very close to *V*_BGC_. A significant shift in *B*_C,NW_ and *B*_C,C_ to smaller values is observed by increasing *V*_BG_ higher than 15 V as illustrated in Figure 4d,e, respectively. Similar dependence with *B* field was observed in Ti-based superconducting nanostructures.^12,15^ We also note that a large scattering in the critical currents values was observed in the measurements at *V*_BG_ > 17 V, which can be attributed to the wide nonthermal switching current distribution as a result of the gating effect.^19,24,39,46^

In previous studies, the origin of GCS was attributed to two different mechanisms, either to the effect of the applied electric field^12−21,25−28^ or to high-energy quasiparticle injection via tunneling.^22−24^ We will compare our experimental findings with these explanations in the following.

GCS has been reported in various device geometries of evaporated polycrystalline metallic nanobridges made of different superconducting materials,^12−21^ and an explanation based on electric field induced distortion of the superconducting wave function that could destroy the BCS state has been proposed,^25−27^ such as, e.g., by Swinger effect.^28^ The observed *B* field and *T* dependencies of the gating effect show characteristics very similar to our epitaxial superconductor case. However, there is a finite leakage current of ≈100 pA at gate voltages where the supercurrent is reduced (see Figure 2a,c). This leakage current is largely *B*-field and temperature independent, as expected (see the Supporting Information). Assuming that the leakage takes place between the gate electrode and the nanowire, in the simplest ballistic picture, hot electrons are injected into the superconducting shell with energies even as high as 10–20 eV, which is several orders of magnitude higher than the SC gap. These electrons could heat up the superconducting bridge and drive it to the normal state, as it is proposed by refs (22−24) as a microscopic origin of the gating effect. Our basic estimation (see the Supporting Information) of induced heat transfer also suggests that the hot electrons could bring the temperature of the epitaxial shell in the range of the superconducting critical temperature. Instead of using silicon dioxide or other amorphous insulators, the gate electrode and the superconductor are separated by a 20–30 nm thick single crystalline hBN layer in our device, which is a large band gap insulator commonly used as a tunnel barrier in 2D electronics.^34,35,38^ Considering a tunnel barrier between the gate and superconductor, the heating effect resulting from hot electron injection should show a strong asymmetric dependence on the polarity of the gate voltage. For the polarity when electrons tunnel from the gate electrode to the superconductor (see Figure 4f (left)), hot electrons relax their energy in the superconductor by inducing a large number of quasiparticles, which results in a significant heat load. On the other hand, for opposite polarity (see Figure 4f (right)), hot electrons heat the metal block of a large gate electrode, which has a much smaller heating effect on the superconductor isolated by the gate insulator. Such gate voltage asymmetry was observed in ref (22) (see Figure 6 in the extended data for the reference). However, it does not appear in our measurements (see Figure 2a) after the initial training period (see the Supporting Information), which contradicts the simple explanation based on ballistic injection of hot electrons. Nevertheless, we should also consider within this comparison the difference in the device geometry between the investigated device (in ref (22)) and the device presented here in our work.

However, if the tunneling process is not ballistic through the hBN, inelastic processes could lead to dissipation within the barrier itself, resulting in more symmetric *I*–*V* curves. These inelastic excitations could most likely be phonons, as explained at the end of this section. However, in suspended nanobridges presented in ref (18), the leakage current and the generation of inelastic excitations are suppressed by orders of magnitude, so the gating effect in these devices could have another origin besides the injection of hot electrons/phonons.

Considering the magnetic field dependence at finite gate voltage (Figure 4d,e), it is consistent with hot electron injection, since increasing the gate voltage leads to an increase in the energy and rate of high-energy electrons injected into the nanowire segment. As a result, the device heats up more and the critical field of the superconducting wire decreases to smaller values. Moreover, as the magnetic field increases, the critical temperature of the superconductor decreases. In turn, a smaller gate voltage could bring the electronic temperature of the device up to this reduced critical temperature of the superconductor. This is visible in Figure 4c, where the reduction of the critical gate voltage is shown.

Finally, we focus on the *T* dependence of *V*_BGC_. In a simple hot electron injection scenario, one would expect that at elevated temperatures, a lower heat load would be sufficient to drive the contact segment to normal state. However, our results show that *V*_BGC_ for the nanowire does not depend on the temperature up to *T*_C,NW_. This alone would be consistent with an electric field-based origin. At temperatures larger than *T*_C,NW_, the nanowire is in the normal state and only the contacts are superconducting. As shown by the red arrow on Figure 3d, *V*_BGC_ decreases at higher temperatures, as expected from Joule heating scenarios. We note that above *T*_C,NW_ the wire itself is already resistive, thus current flow induces additional dissipation, which could also contribute.

In summary, the temperature dependence cannot be described by a simple ballistic injection of hot electrons alone, but the strong dependence on the leakage current also makes the origin due to an electric field unlikely.

At the end we note a parallel work of Ritter et al.,^39^ in which the authors investigated the origin of the gating effect on TiN nanowires. They found that the decrease in supercurrent for their devices was independent of the electric field between the wire and the gate electrode. The suppression of the supercurrent was attributed to the generation of non-equilibrium phonons due to relaxation of high-energy electrons in the substrate. The generated phonons could propagate through the substrate over distances that exceed 1 μm. Once these high-energy phonons reach to the superconducting device, they generate a large number of quasiparticles and suppress the supercurrent. A similar scenario is likely to be present in our device, where the phonons spread through the hBN layer and suppress the supercurrent in the nanowire and contact segments. Moreover, this scenario explains, for example, the symmetry of GCS with gate voltage present in our measurements. The observed magnetic field and temperature dependencies are also compatible with heat transfer via phonons.

To sum up, the phonon generation scenario might explain most of our findings; however, it is likely that the origin of the GCS effect depends heavily on the device architecture, since, e.g., for suspended devices of ref (18), another origin might be present. In order to achieve reliable, fast, and integrated superconducting electronics based on GCS, further detailed studies on the various existing experimental platforms are required.

## Conclusions

In summary, we have demonstrated the superconducting gating effect in an epitaxially grown superconducting layer for the first time. We developed a novel gate-controlled supercurrent transistor using an Al shell around an InAs nanowire, which acts as the active region. The device shows a full suppression of the supercurrent by applying ≃±23 V on a bottom gate insulated from the nanowire by a high-quality single crystalline hBN layer. Detailed magnetic field and temperature dependent characterization allowed us to compare the experimental facts with existing scenarios of superconducting gating. The gating effect independent of the polarity suggests that the simple ballistic hot electron injection does not provide a complete explanation of the observed gating. However, the strong correlation between the suppression of the critical current and the increase in the leakage current suggests that the electric field is unlikely an origin of the gating effect in our particular device. Phonon generation in the tunnel barriers, however, can give an explanation which is consistent with most of our findings. Besides the fundamental interest, our results open the way to integrate superconducting switches into novel Al/InAs-based hybrid quantum architectures.

## Supporting Information Available

Methods used in device fabrication and experiments, measurement of the other pair of the contacts, correction of the leakage current, correlation between the leakage current and the critical current, magnetic field dependence of the nanowire segment, influence of temperature and magnetic field on the leakage current, theoretical model, and initial training measurements (PDF)
