# Supplementary material for: Gate-Controlled Supercurrent in Epitaxial Al/InAs Nanowires
Source: Nano Lett. 2021 Nov 2;21(22):9684–90. doi: 10.1021/acs.nanolett.1c03493 (PMC8631737; doi:10.1021/acs.nanolett.1c03493)
Supplement: Supplementary file 1 — nl1c03493_si_001.pdf [file nl1c03493_si_001.pdf]

# Gate-controlled supercurrent in epitaxial Al/InAs nanowires

Tosson Elalaily,[1,2,*] Olivér Kürtössy,[1,*] Zoltán Scherübl,[1,3] Martin Berke,[1] Gergő Fülöp,[1] István Endre Lukács,[4] Thomas Kanne,[5] Jesper Nygård,[5] Kenji Watanabe,[6] Takashi Taniguchi,[7] Péter Makk,[1,†] and Szabolcs Csonka[1,‡]

[1]*Department of Physics, Budapest University of Technology and Economics and Nanoelectronics 'Momentum' Research Group of the Hungarian Academy of Sciences, Budafoki ut 8, 1111 Budapest, Hungary*

[2]*Department of Physics , Faculty of Science, Tanta University, Al-Geish St., 31527 Tanta, Gharbia, Egypt.*

[3]*Univ. Grenoble Alpes, CEA, Grenoble INP, IRIG, PHELIQS, 38000 Grenoble, France*

[4]*Center for Energy Research, Institute of Technical Physics and Material Science, Konkoly-Thege Miklós út 29-33., H-1121, Budapest, Hungary*

[5]*Center for Quantum Devices and Nano-Science Center, Niels Bohr Institute, University of Copenhagen, Universitetsparken 5, DK-2100, Copenhagen, Denmark*

[6]*Research Center for Functional Materials, National Institute for Material Science, 1-1 Namiki, Tsukuba, 305-0044, Japan*

[7]*International Center for Materials Nanoarchitectonics, National Institute for Materials Science, 1-1 Namiki, Tsukuba 305-0044, Japan*

## I. METHODS

InAs nanowires with 20 nm thick Al shell and total diameters of 82-162 nm were grown by Au-assisted molecular beam epitaxy (MBE). After InAs nanowire growth with typical lengths of 2.7-6 $\mu$m, the Al shell layer was epitaxially grown around the nanowire by rotating the substrate and depositing at an angle within the MBE chamber at low temperature. Based on TEM characterization, the Al is highly crystalline, containing only a few grain boundaries on the length of our device[1].

The device was fabricated by electron beam lithography (EBL) in two separate steps. In the first step, metallic gates of Ti/Au layers with thicknesses of 7/33 nm and width of 600 nm were fabricated on an intrinsic Si wafer with a 290 nm thick oxide layer. hBN flakes were mechanically exfoliated from the bulk onto a clean 290 nm thick $SiO_2$/Si wafer by using adhesive tape. A polydimethylsiloxane (PDMS) polymer stamp prepared with polycarbonate (PC) layer was used to pick up the hBN flakes at 75°C with a thickness of 20-30 nm, which can be identified from their contrast on the Si wafer under an optical microscope. The flakes are then transferred and released on the top of the bottom gates by melting the PC layer at 120°C. PC layer was then dissolved by left the substrate in chloroform for 20 minutes[2–5]. After drying the substrate, nanowires were deposited on the top of the hBN layer[6,7] and aligned perpendicular to the bottom gate by a thin glass needle with a microscopic tip controlled by a hydraulic micromanipulator along with a high magnification optical microscope. Finally, four contacts of Ti/Al with thicknesses of 10/80 nm were fabricated in the second lithography step. Before this last evaporation step, nanowires were exposed to Ar-ion plasma milling for 8 minutes at 50 W to remove any oxides on the top of Al shell[8,9].

All measurements were carried out on Leiden Cryogenics CF-400 top loading cryo-free dilution refrigerator system with a base temperature of 30 mK. We used a standard power supply with a series 1 MΩ resistor to inject current through the nanowire via a pair of Al contacts on opposite sides of the nanowire and measured the voltage across the other pair with a voltage differential amplifier. The leakage current was recorded by measuring the voltage across 10 MΩ preresistor connected to the gate. The I-V characteristics of the device were measured using a quasi 4-probe method (see Fig. 1a in the main text) to exclude the resistance of the electrodes. Therefore, the only part of the electrodes that contributes to

the 4-probe conductance is the small superconducting piece on top of the wire, which results in the switching at 2.34 $\mu$A. The origin of this switching is confirmed by measurements between electrodes at the same end of the wire using the 2-probe method.

## II. MEASUREMENTS OF THE OTHER PAIR OF CONNECTED AL CONTACTS

In the main text, we have shown the IV characteristics of one of the contacts, here, in SFig. 1 the IV characteristics of the other pair of Al contacts measured by the 2-probe method is presented. The measurement shows switching of these contacts from the superconducting state to normal state at $\pm$ 7.5 $\mu$A which is larger than the value of both $I_{\mathrm{C,NW}}$ and $I_{\mathrm{C,C}}$ in the IV characteristics of the nanowire device in the main text.

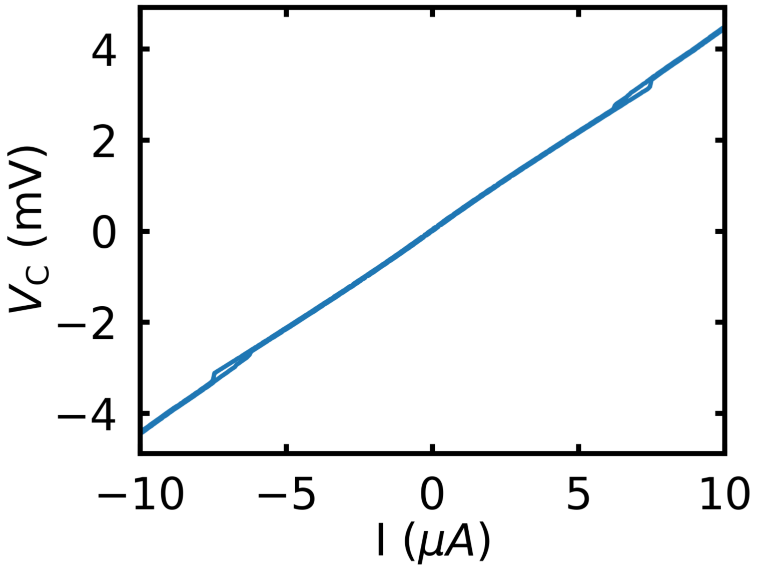


SFig. 1. IV characteristics of the other pair of connected Al contacts measured by 2-probe method.

## III. CORRECTION OF THE LEAKAGE CURRENT MEASUREMENTS

The leakage current measured by the preresistor connected to the gate during investigation of the device is shown in the red curve in SFig. 2 and contains two current components: a leakage current from the gate to the nanowire device plus other leakage components e.g. through the wiring of the measurement setup. To extract the exact leakage current of the investigated device, the latter component should be subtracted from the measurements in

the entire gate voltage range. For that purpose, we have connected one contact of the device to an I/V converter and let the other contacts on float. By sweeping the gate voltage up to $\pm V_{BGC}$, the current measured through the I/V converter would give only the leakage current component to the nanowire device (green curve)[10]. As SFig. 2 shows there is a difference between the current leaking from the gate (preresistor) and the current drained by the superconducting nanowire (I/V conv). Since the latter current can not be measured during supercurrent measurement we estimated the leakage current to the superconducting nanowire in the following way: from the leakage current measured by the preresistor the difference between the linear slope of the preresistor measurement (blue dotted line) and I/V converter measurement (black dotted line) was subtracted. These leakage current values are plotted in Fig. 2c in the main text.

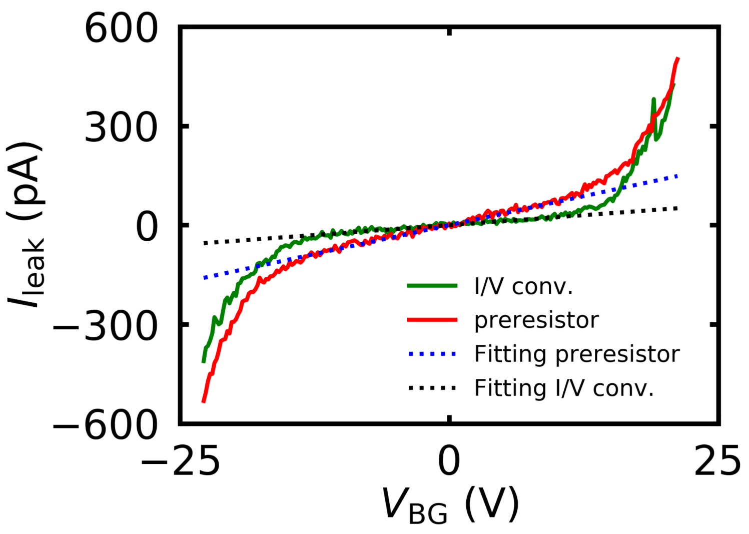


SFig. 2. Leakage current measurements by preresistor (red curve) and I/V converter (green curve). The two doted lines are linear fit to the I/V converter (black) and prepresistor (blue) based leakage currents for small gate voltage values.

## IV. CORRELATION BETWEEN THE LEAKAGE CURRENT AND THE CRITICAL CURRENT

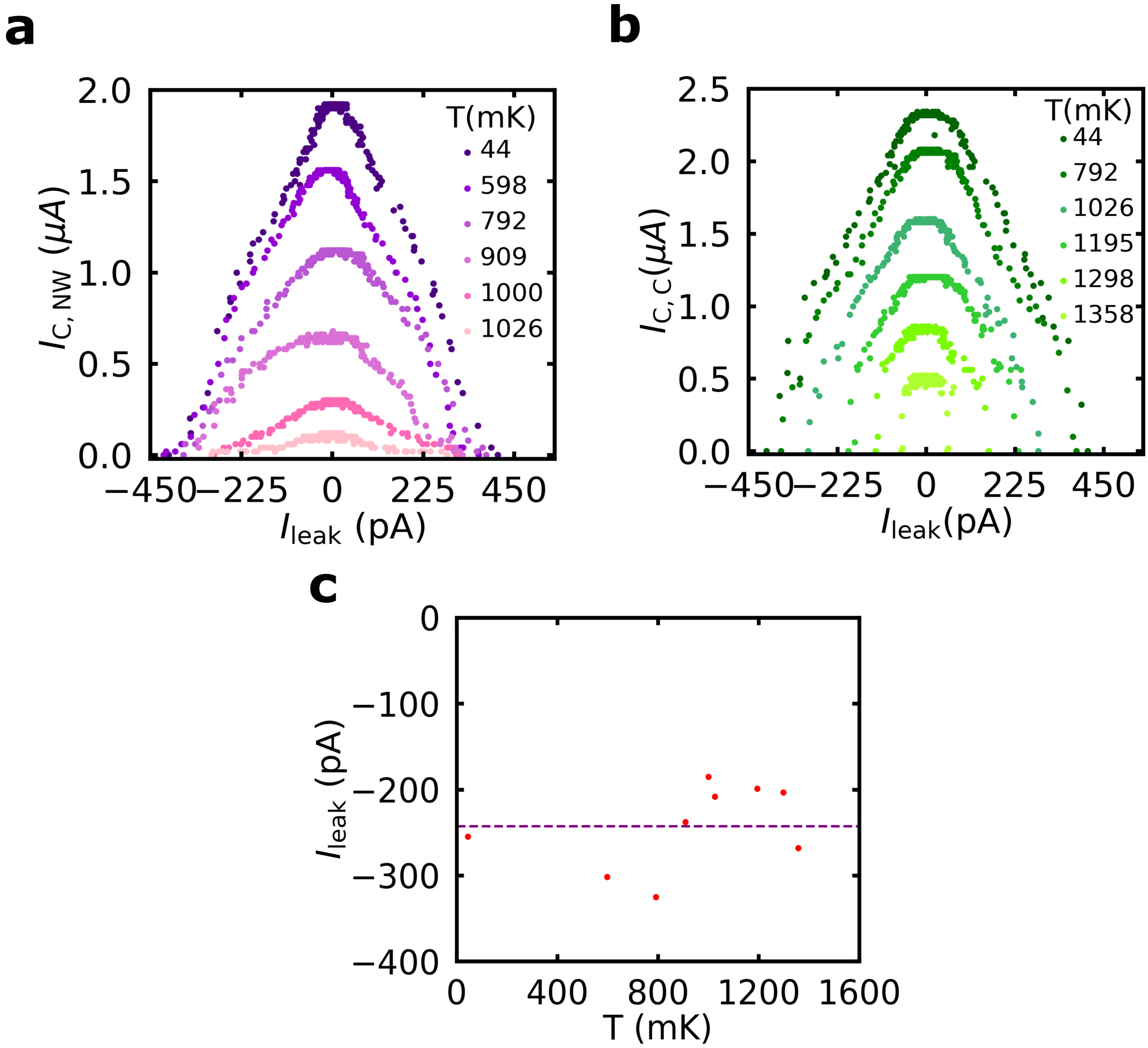


SFig. 3. Parametric curve between $I_C$ and $I_{leak}$ at elevated temperatures for **(a)** nanowire segment **(b)** contact segment.**(c)** The average leakage current within 1 V window at $V_{BG}$ = -21 V is plotted as a function of the bath temperature, while the purple dashed line represents the mean value of the plotted data.

In Fig. 3c,d in the main text, $I_C$ was plotted as a function of $V_{BG}$. The observed supercurrent suppression strongly correlates with enhancement of the leakage current as a function of gate voltage (see Fig. 2a and c in the main text), thus we present the critical

current as a function of the leakage current as well. Such parametric $I_C$ vs $I_{leak}$ curves are shown in SFig. 3a,b. The plotted curves are symmetric and similar to what observed in Ref. 10, where the gating effect is attributed to the injection of hot electrons. They also show that the critical currents start to decrease e.g. at the base temperature at around 35 pA and get fully suppressed around 410 pA.

We note that, a more careful inspection of these curves show that the shape of these curves depends on temperature (e.g. the critical leakage current where the supercurrent is fully suppressed), however, this deceptive dependence originates from temporal fluctuation of the leakage current, since the measurements presented in SFig. 3a, b were carried out in several hours time window. In SFig. 3c the leakage current is plotted around a fixed gate voltage ($V_{BG}$= -21 V) averaged in 1 V window. $I_{leak}$ shows random dependence on T with a fluctuation more than 50 % of the mean value. This temporal change of the leakage current contributes to the irregular, unique shape of the curves in SFig. 3a, b.

## V. MAGNETIC FIELD DEPENDENCE OF NANOWIRE SEGMENT

SFig. 4 shows the GCS dependence of the critical current of the nanowire segment $I_{C,NW}$ under influence of the external magnetic field B. Since the observed dependence of $V_{BGC}$ on B is strong at field values very close to the critical field, the magnetic field dependence of $I_{C,NW}$ shows a weaker dependence compared to that of the contact segment $I_{C,C}$ (Fig. 4c in the main text) because the measurements are carried out only up to 46 mT which is a bit far from the critical field value of nanowire segment $B_{C,NW}$ = 66 mT, while for the contact segment $B_{C,C}$ = 50 mT.

## VI. INFLUENCE OF TEMPERATURE AND MAGNETIC FIELD ON THE LEAKAGE CURRENT

The temperature and magnetic field dependencies of the leakage current measured during investigation of the device are shown in SFig. 5a,b, respectively after correction with the method explained in section III. The measurements show that the measured leakage current is independent of either the elevated temperatures or the external magnetic field.

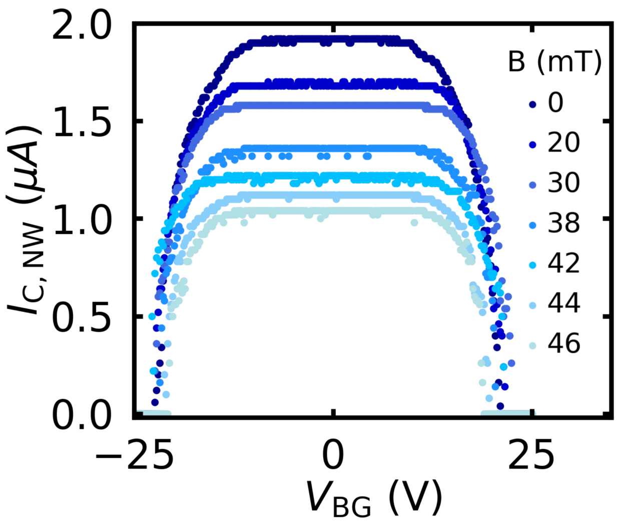


SFig. 4. $I_{\mathrm{C,NW}}$ as a function of bipolar gate voltage at magnetic fields up to 46 mT.

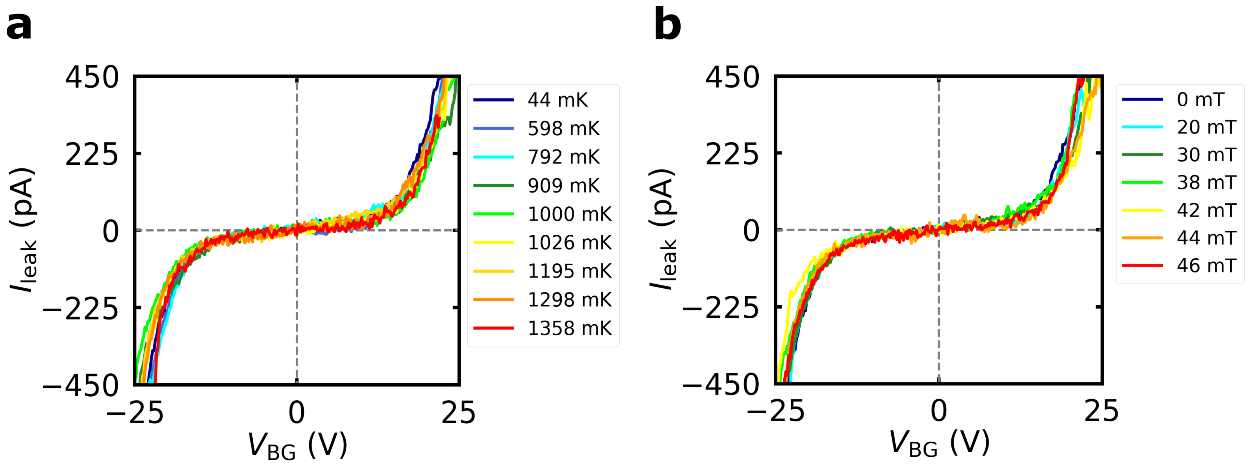


SFig. 5. **(a)** The leakage current $I_{\mathrm{leak}}$ as a function of the gate voltage $V_{\mathrm{BG}}$ at elevated temperatures and **(b)** at different values of external magnetic field.

## VII. THERMAL MODEL FOR CALCULATING THE ELECTRONIC TEMPERATURE OF NANOWIRE DEVICE

In this section, we will give a simple estimation for the increase of the electronic temperature of the nanowire segment resulting from the injection of hot electrons from the gate. Suppose that a single electron tunnels from the gate electrode to the Al layer (that has a volume $\Omega$) with energy $eV_{\mathrm{BG}}$, that is much higher than the superconducting gap of Al as illustrated in the schematic in SFig. 6a. This electron will release its energy to the electron

gas. Assuming that the temperature of the Al shell is initially at temperature $T_\mathrm{i}$, the injection of an electron to the Al shell will result in a sharp increase on its electronic temperature derived from the heat equation:[11,12]

$$T_{i+1} = \sqrt{\frac{2eV_\mathrm{BG}}{\Omega\gamma} + T_\mathrm{i}^2}, \tag{1}$$

where $\gamma$ is the Sommerfeld coefficient. The temperature difference between the nanowire segment and the contacts which are at base temperature $T_\mathrm{b}$ = 100 mK will result in a cool-down of the nanowire segment through heat diffusion by electrons to the contacts. The amount of heat transferred within time $dt$ is given by:

$$Q = \frac{KA\Delta T}{L} \cdot dt, \tag{2}$$

where the Wiedemann-Franz law is used:

$$K = \sigma \mathscr{L} T_i, \tag{3}$$

where K is the thermal conductivity of the Al shell, $\sigma = R_\mathrm{n}L/A$ is its electrical conductivity, $L$ is the half length of the nanowire segment from the injection point to the contacts, $A$ is its cross-sectional area, $R_n$ is its normal state resistance, $\mathscr{L}$ is the Lorentz number and $\Delta T$ is the temperature difference between actual temperature of the middle of the nanowire, $T_i$ and the temperature of the contact $T_\mathrm{b}$: $\Delta T = T_\mathrm{i}$ - $T_\mathrm{b}$. Due to the heat loss to the contacts, the electronic temperature of the nanowire segment $T_i$ will be decreased to $T_{i+1}$ given by the following heat loss equation:

$$T_{i+1} = T_i - \frac{Q}{\Omega\gamma T_i}. \tag{4}$$

Assuming that at every time period of $t_\mathrm{inj} = e/I_\mathrm{leak}$ (where $I_\mathrm{leak}$ is the leakage current shown in Fig. 2c in the main text) a hot electron arrives and in the meantime the wire continuously cools, the time dependent temperature can be simulated by Eqs. 1 - 4 in discretized points $T_\mathrm{i}$, $i$=1, 2, 3, ....

SFig. 6b shows the calculated temperature as a function of time, for a time period that contains 30 successive electron injection cycles ($t_{inj}$) at different gate voltages by assuming that the total diameter of the nanowire = 100 nm, and using $\Omega$ = 7.536 $\times$ $10^{-21}$ m$^3$ ,$\gamma$ = 135.1 Jm$^{-3}$K$^{-2}$, $R_\mathrm{n}$= 135 $\Omega$, $A$= 5.024 $\times$ $10^{-15}$ $m^2$ and $\mathscr{L}$= 2.45 $\times$ $10^{-8}$ $V^2$ $K^{-2}$. At smaller gate voltages, the leakage current is quite small and the corresponding electron

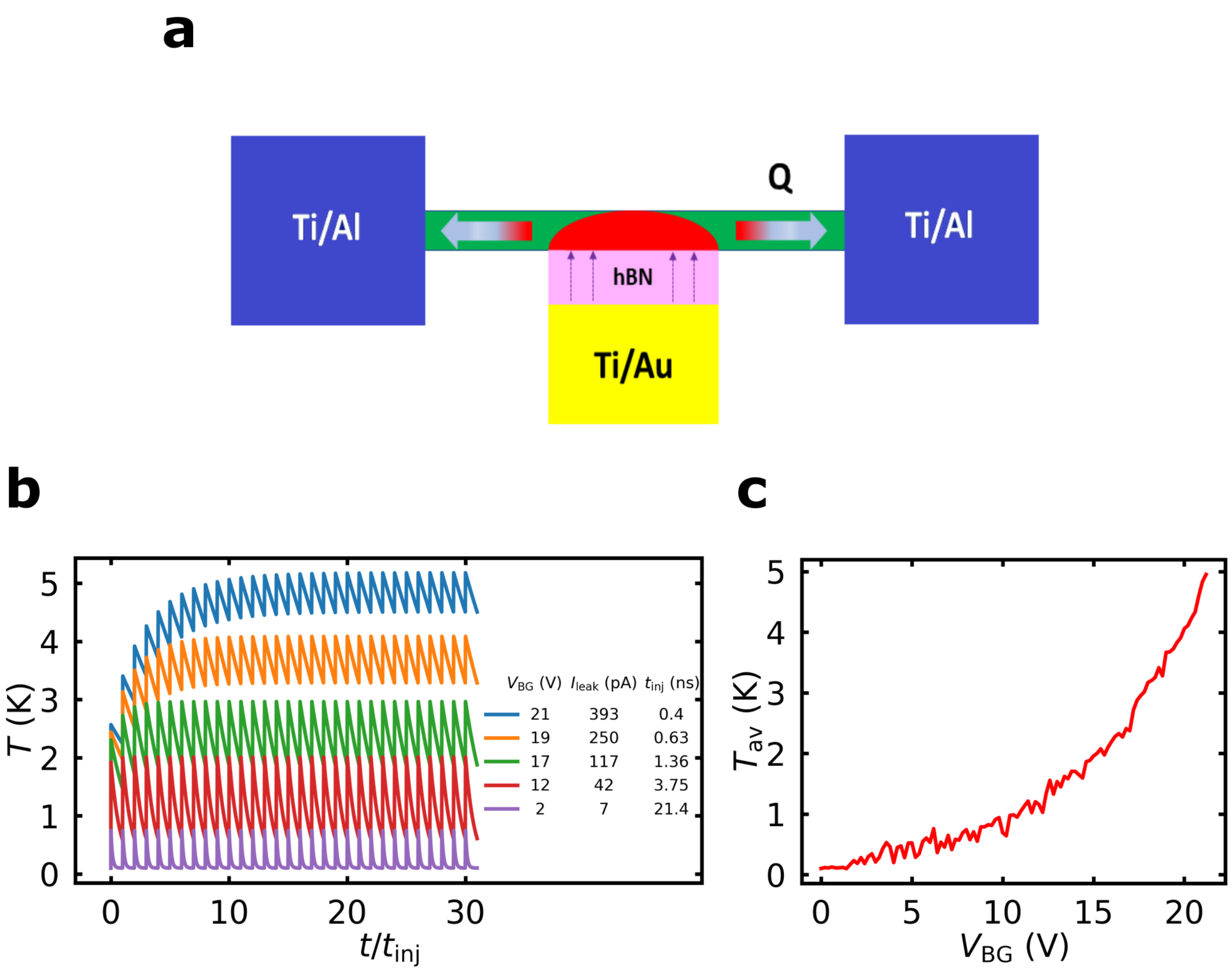


SFig. 6. **(a)** Schematic of the thermal model for calculating the electronic temperature of the nanowire device after injection of electrons with energy $eV_g$ from the gate electrode. **(b)** Calculated electronic temperature of the nanowire segment within 30 successive electron injection cycles at different gate voltages, where $t_{inj} = e/I_{leak}$.**(c)** The average nanowire temperature over 20 injection cycles (taken at the temperature saturation region) as a function of gate voltage.

injection time $t_{inj}$ is very long (see table in SFig. 6b), the nanowire segment will cool down to base temperature before the next electron is injected (see purple curve in SFig. 6b), while at larger gate voltages, $t_{inj}$ is very small and the system could not cool down close to base temperature before the next electron is injected. In the latter case, the successive injections will increase the effective temperature of the nanowire until it reaches a steady state temperature (see blue curve SFig. 6b). SFig. 6c shows the average nanowire temperature (taken over 20 injection cycles at the saturation region) as a function of gate voltage. The calculated temperature increases exponentially with increasing gate voltage up to val-

ues in the same order of magnitude as the critical temperatures $T_{\mathrm{C,NW}} = \simeq 1050$ mK and $T_{\mathrm{C,C}} \simeq 1400$ mK. The above calculation is based on some simplifications, like the heat transfer via phonons is neglected, the wire is handled as a normal conductor. Based on the above simple estimation, leakage current-based suppression of the supercurrent could be a feasible explanation of superconducting gating, since the heat transfer of hot electrons could bring the superconducting nanobridge to normal state.

## VIII. INITIAL TRAINING MEASUREMENTS ON NANOWIRE DEVICE

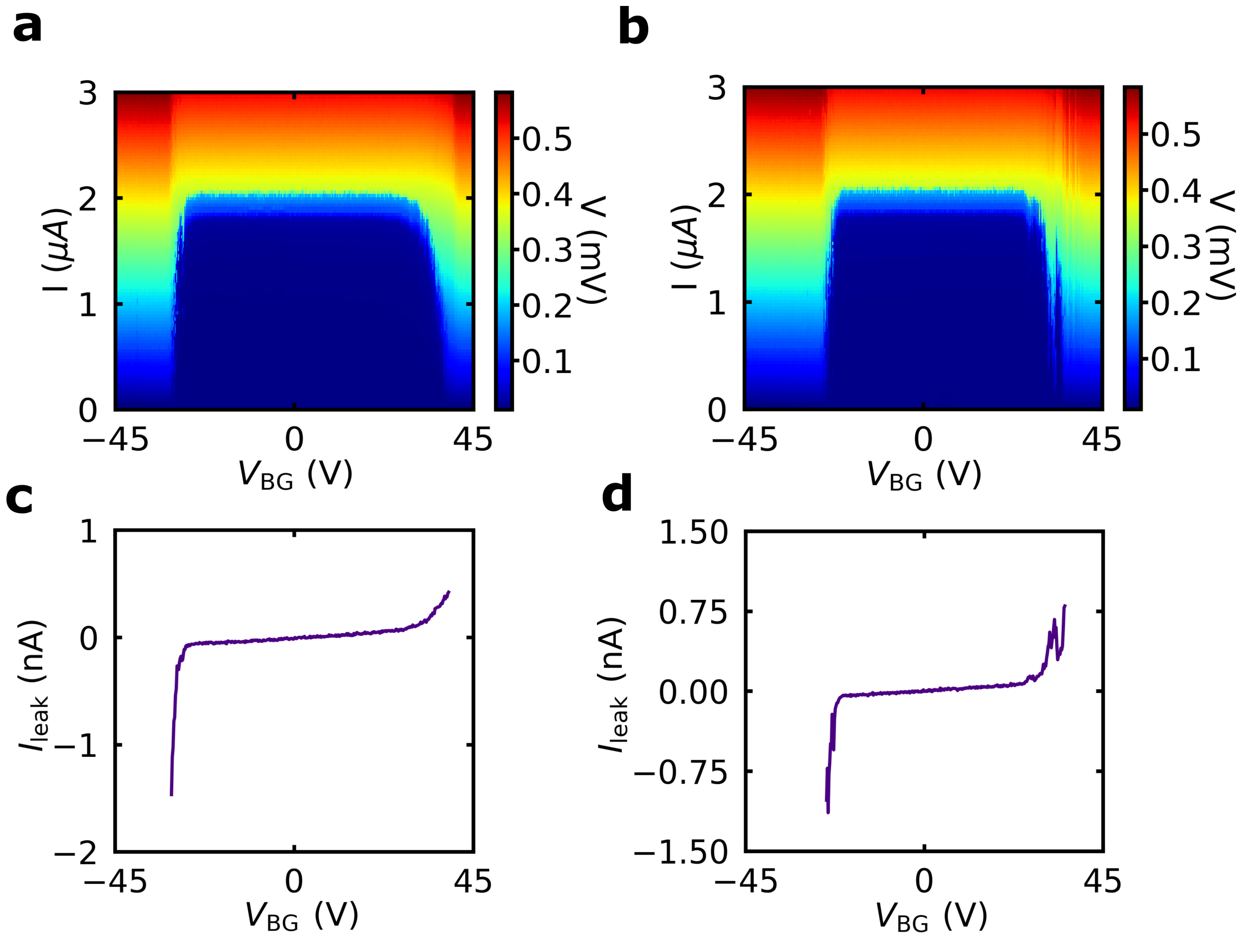


SFig. 7. **(a)**,**(b)** IV characteristics of nanowire device as a function of bipolar gate voltage $V_{\mathrm{BG}}$ for two successive measurements and thier corresponding leakage current in **(c)**,**(d)**, respectively.

Investigation of the gating effect at the earlier training measurements on the nanowire device shows asymmetry on the supercurrent-gate voltage dependence and the device switches

to normal state at higher gate voltage (see SFig. 7a) compared to symmetric measurements in Fig. 2a in the main text. The asymmetry on the supercurrent dependence is accompanied by a similar asymmetry on the leakage current recorded during the measurements as shown in SFig. 7c. By repeating the measurements, the supercurrent dependence and the corresponding leakage current becomes more symmetric on the bipolar gate voltage (see SFig. 7 b,d, respectively) and the critical gate voltage $V_{\mathrm{BGC}}$ is decreased to lower values. Ramping the gate voltage back and forth many times leads to symmetric measurements shown in the main text.

The initial training period of the gate dependence of the switching current suggests that at the beginning there might be initial changes happening either on the surface of the superconductor or in the insulator barrier itself. Similar training cycles are seen in the electroformation of memristors, where often the crystalline structure of the barrier e.g. $SiO_2$ changes[13]. Another explanation could be filling of the neighbouring charge traps present e.g. in the hBN barrier.

---

* Contributed equally to this work

† makk.peter@ttk.bme.hu

‡ szabolcs.csonka@ttk.bme.hu

1 P. Krogstrup, N. Ziino, W. Chang, S. Albrecht, M. Madsen, E. Johnson, J. Nygård, C. M. Marcus, and T. Jespersen, Nature materials **14**, 400 (2015).

2 L. Wang, I. Meric, P. Huang, Q. Gao, Y. Gao, H. Tran, T. Taniguchi, K. Watanabe, L. Campos, D. Muller, *et al.*, Science **342**, 614 (2013).

3 P. Zomer, M. Guimarães, J. Brant, N. Tombros, and B. Van Wees, Applied Physics Letters **105**, 013101 (2014).

4 M. Gurram, S. Omar, S. Zihlmann, P. Makk, C. Schönenberger, and B. Van Wees, Physical review B **93**, 115441 (2016).

5 F. Barati, J. P. Thompson, M. C. Dartiailh, K. Sardashti, W. Mayer, J. Yuan, K. Wickramasinghe, K. Watanabe, T. Taniguchi, H. Churchill, and J. Shabani, Nano Letters **21**, 1915 (2021).

6 Z. Scherübl, A. Pályi, G. Frank, I. E. Lukács, G. Fülöp, B. Fülöp, J. Nygård, K. Watanabe,

T. Taniguchi, G. Zaránd, and S. Csonka, Communications Physics **2**, 1 (2019).

[7] F. Jekat, B. Pestka, D. Car, S. Gazibegović, K. Flöhr, S. Heedt, J. Schubert, M. Liebmann, E. P. Bakkers, T. Schäpers, and M. Morgenstern, Applied Physics Letters **116**, 253101 (2020).

[8] W. Chang, S. Albrecht, T. Jespersen, F. Kuemmeth, P. Krogstrup, J. Nygård, and C. M. Marcus, Nature nanotechnology **10**, 232 (2015).

[9] S. Vaitiekėnas, Y. Liu, P. Krogstrup, and C. Marcus, Nature Physics , 1 (2020).

[10] M. Ritter, A. Fuhrer, D. Haxell, S. Hart, P. Gumann, H. Riel, and F. Nichele, Nature communications **12**, 1 (2021).

[11] M. Rocci, G. De Simoni, C. Puglia, D. D. Esposti, E. Strambini, V. Zannier, L. Sorba, and F. Giazotto, ACS nano **14**, 12621 (2020).

[12] C. Puglia, G. De Simoni, and F. Giazotto, Physical Review Applied **13**, 054026 (2020).

[13] L. Pósa, M. El Abbassi, P. Makk, B. Sánta, C. Nef, M. Csontos, M. Calame, and A. Halbritter, Nano letters **17**, 6783 (2017).
